# A Rare Instance of Chronic Masson’s Tumor Presenting in the External Auditory Meatus and Surgical Plan: A Case Report

**DOI:** 10.7759/cureus.50314

**Published:** 2023-12-11

**Authors:** Feras Othman, Sadhana Anantha, Gavin H Ward, Elizabeth Paulus, Rita Romaguera

**Affiliations:** 1 General Surgery, University of Miami, Miami, USA; 2 School of Medicine, St. George's University School of Medicine, True Blue, GRD; 3 Pathology, University of Miami, Miami, USA

**Keywords:** pathology, ent, intravascular papillary endothelial hyperplasia, surgery, masson’s tumor

## Abstract

Intravascular papillary endothelial hyperplasia (IPEH), or Masson’s tumor, is a rare and benign proliferation of endothelial cells typically of vascular origin. Common locations of Masson’s tumor include the head, neck, orbit, lip, pharynx, and mandible. It is typically seen in middle-aged adult life and females. Possible differential diagnoses include hemangioma, benign vascular formation, angiosarcoma, and neurofibromatosis. The exact pathophysiology of Masson’s tumor is currently unknown. We present the case of a middle-aged 47-year-old male with a pure type of Masson’s tumor presenting with pedunculated, malleable lesions across the posterior scalp and circumferential neck, on the pinna of the right ear, and within the right external auditory meatus. The lesions within the right external auditory meatus caused conductive hearing loss. The plan is a complete surgical excision without wide margins. The patient was referred to an ear, nose, and throat (ENT) surgeon due to the complicated location of the lesion within the external auditory meatus. This case serves as a differential diagnosis of conductive hearing loss complicated by Masson's tumor.

## Introduction

Intravascular papillary endothelial hyperplasia (IPEH), or Masson’s tumor, is a benign proliferation of endothelial cells typically limited to the vascular lumen [1]. Due to overlapping histologic features, IPEH can initially be misdiagnosed as a malignancy such as angiosarcoma or Kaposi’s sarcoma [2,3]. The pathogenesis of this tumor is poorly understood. Frequently presenting in a vessel, IPEH is not a primary vascular tumor on pathological analysis but rather a benign lesion of vascular origin characterized by abnormal proliferation of endothelial cells [4].

Common locations include the head, neck, orbit, lip, pharynx, and mandible [5,6]. It is generally seen in the fourth decade of life but can be seen in the second to sixth decades of life [3]. It is more common in females. It is not clear why it is more common in adult life and females. To the best of our knowledge, the localization of IPEH invading the external auditory meatus is not reported in literature. Vascular tumors of the external auditory meatus frequently were categorized as capillary hemangiomas [7,8]. Presentation is typically dependent on the location, which is ideally characterized by a magnetic resonance imaging (MRI) scan [9]. The treatment of choice is complete surgical resection without the need for wide margins due to the low probability of recurrence [10]. The plan for the patient presented in our case is a referral to an ears, nose, and throat surgeon for surgical excision without wide margins. Due to the complex location of the patient's IPEH in the external auditory canal, it may be difficult to remove all of the tumor growth. However, the advantages of the surgery are an improvement in the patient's conductive hearing and a relatively low chance of recurrence. Aggressive forms of IPEH have a recurrence rate of up to 10%, and if the IPEH is recurring, then chemotherapy and radiation may be utilized [10]. The broader implications of this case are IPEH as a potential differential diagnosis for conducting hearing loss. 

## Case presentation

Patient information

A 47-year-old man with no pertinent past medical history presented to the outpatient clinic in July of 2022 with multiple polypoid lesions in the head, neck, and upper chest. 

Timeline

**Figure 1:**
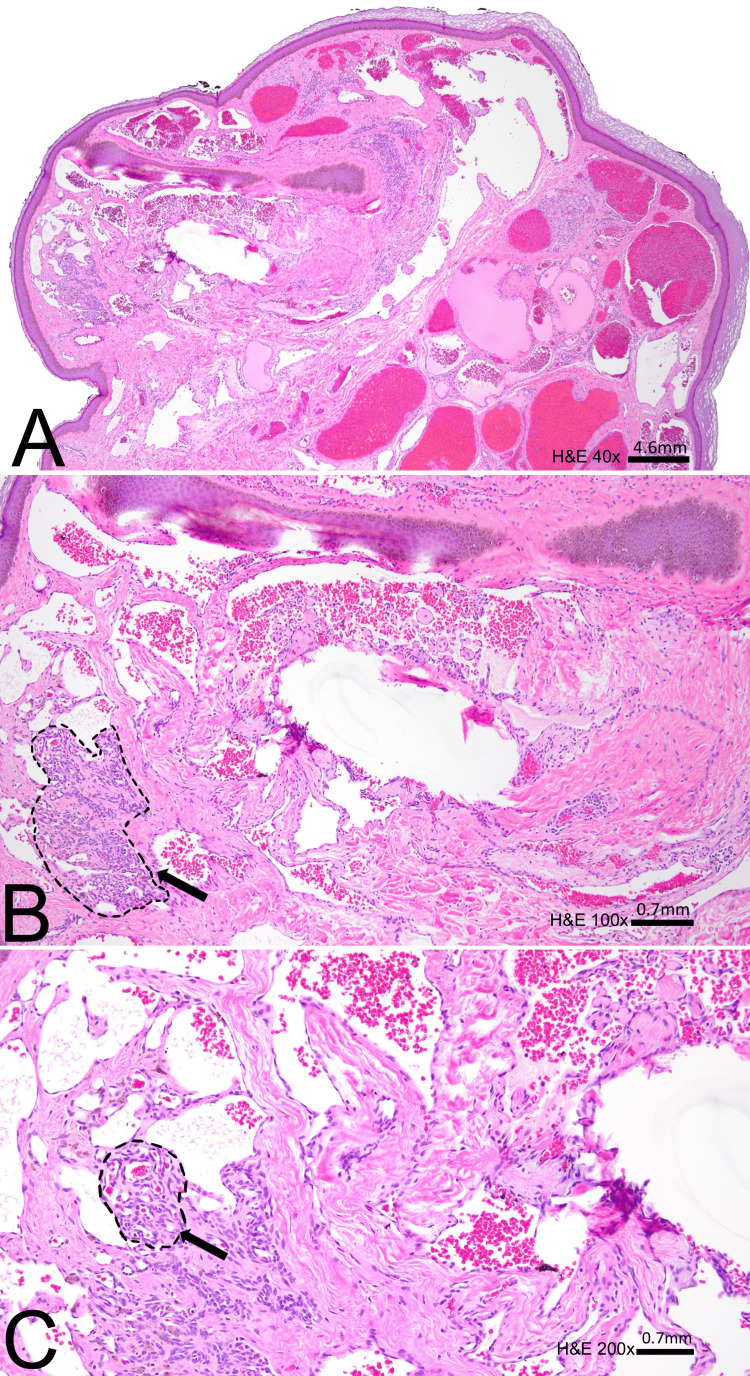
(A) H&E 40x slide of polypoid skin lesion with subepithelial dermal dilated vascular proliferation; (B) H&E 100x slide of subepithelial dermal dilated vascular proliferation with focal papillary endovascular proliferation (arrow); (C) H&E 200x slide of dermal dilated vascular proliferation with focal endovascular papillary proliferation of endothelial cells with a fibrovascular core (arrow). H&E: hematoxylin and eosin.

He was previously seen in the clinic in 2009 for lesions he has had since 25 years old. He saw multiple specialists over the years and was repetitively reassured his lesions were moles. During his clinic visit with us in 2022, his clinical exam demonstrated widespread soft polyps across his head and neck akin to a neurofibromatosis (NF) presentation. A few lesions were excised and sent to pathology. Pathology demonstrated the polyps were categorized as hemangiomas, specifically intravascular papillary growth pathognomonic for Masson’s tumor (Figure 1).

The patient presented at a later date to the clinic for the rapid growth of new lesions along his head and neck and recently decreased hearing acuity in his right ear for the past few months.

Clinical findings

**Figure 2:**
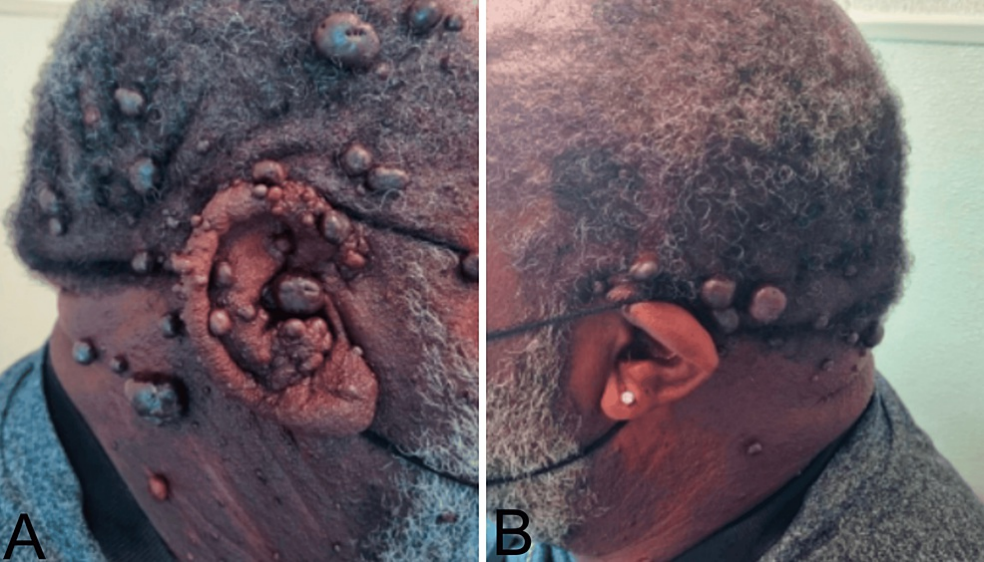
Physical examination of the scalp, (A) right ear, and (B) left ear demonstrating the patient's IPEH. IPEH: intravascular papillary endothelial hyperplasia.

Clinical examination revealed pedunculated, malleable lesions across the head and neck and numerous lesions outside of the right ear and within the external auditory meatus. On the left ear, lesions were only noticed on the outside of the pinna following into the scalp (Figure 2).

All the lesions were polyploidy and soft, with no tenderness on palpation and no signs of inflammation. Systems review revealed no additional sensory, motor, or autonomic symptoms. No additional sensory impairment or numbness was expressed on palpation of the lesions. The patient had no history of lesions, genetic disorders in his family, or prior surgeries. Social and occupational history was non-contributory.

Diagnostic assessment

The patient was referred to an ear, nose, and throat (ENT) surgeon to determine the etiology of the right hearing loss. Masson lesions were determined to be the cause of conductive hearing loss, and the ENT surgeon recommended surgical excision. The patient received a computed tomography (CT) brain scan, which demonstrated normal findings. Genetic testing came back negative for neurofibromatosis (NF).

Therapeutic intervention

Treatment of IPEH is complete surgical excision without wide margins; however, recurrence is possible if the tumor is not completely resected [11]. The patient might be referred to a different service for surgery based on the location of the tumor in our case. The patient was referred to an ENT surgeon because of the involvement of the external auditory meatus.

## Discussion

In our case, the diagnosis of IPEH was prolonged due to a wide range of differential diagnoses and a lack of histological analysis. The tumor is known to mimic common entities such as hemangiomas and vascular formations [3]. Clinically, IPEHs usually present as well-defined, superficial papules that are red to purple in color. They are small in size, measuring 0.5 to 5 cm. They usually grow in the skin, dermis, or subcutaneous tissues [6,8].

Angiosarcoma is an important differential diagnosis to consider due to its malignancy [7]. Angiosarcoma is distinguished from IPEH by the presence, at the histopathological examination, of solid and necrotic areas, mitotic figures, or pleomorphic cells and by the absence of a confined intralaminar location [12,13]. There are no malignant features in our pathologic analysis. In our case, the clusters and pattern of masses indicated the differential diagnosis of neurofibromatosis.

There are three distinct types of IPEH; pure (55.8%) arises de novo; mixed (39.9%) are found superimposed over a preexisting vascular anomaly such as arteriovenous malformations and hemangiomas; and extravascular (4.3%) is associated primarily with trauma-induced hematoma formation [14]. Our patient had no history of trauma, arteriovenous malformations, or hemangiomas, therefore presenting as the most predominant case of pure, de novo, IPEH.

The exact pathophysiology of IPEH is currently unknown; however, the variety among IPEH serves to aid histological diagnosis. Surgical excision is the treatment of choice, but embolization of the feeding vessels has been successful in some cases [10]. Additionally, recurrence rates are relatively low, and only in aggressive cases of IPEH are recurrence rates up to 10%. If the IPEH does recur, then chemotherapy and radiation treatment may be deployed, which have shown success in the management of IPEH.

Our patient's primary complication is conductive hearing loss due to IPEH within the right external auditory meatus. There is no involvement of cranial nerves V3, VII, or the auricular branch of X, as the patient's sensory and motor functions were intact [15]. The patient's IPEH did not involve extrinsic ear muscles, such as auricularis superior, anterior, and posterior. The auricle's blood supply is supplied by the superior temporal artery and posterior auricular artery via the external carotid artery.

## Conclusions

This case report emphasizes the possible relevance and importance of a pure, de novo, IPEH as a possible cause of conductive hearing loss. The appropriate treatment is a complete surgical excision without wide margins. If the IPEH does recur, then chemotherapy and radiation treatments can be explored. IPEH should be noted as a rare differential diagnosis for conductive hearing loss. Future studies should explore other types of IPEH, such as mixed and extravascular, as possible causes for IPEH to occur within the external auditory meatus and cause conducting hearing loss. This case contributes an example of de novo IPEH as a cause of conductive hearing loss due to tumor growth within the external auditory meatus. 
